# Biochemical and Proteomic Analysis of Ubiquitination of Hsc70 and Hsp70 by the E3 Ligase CHIP

**DOI:** 10.1371/journal.pone.0128240

**Published:** 2015-05-26

**Authors:** Sarah E. Soss, Kristie L. Rose, Salisha Hill, Sophie Jouan, Walter J. Chazin

**Affiliations:** 1 Department of Biochemistry, Vanderbilt University, Nashville, Tennessee, United States of America; 2 Center for Structural Biology, Vanderbilt University, Nashville, Tennessee, United States of America; 3 Mass Spectrometry Research Center, Vanderbilt University, Nashville, Tennessee, United States of America; 4 Department of Chemistry, Vanderbilt University, Nashville, Tennessee, United States of America; UMCG, NETHERLANDS

## Abstract

The E3 ubiquitin ligase CHIP is involved in protein triage, serving as a co-chaperone for refolding as well as catalyzing ubiquitination of substrates. CHIP functions with both the stress induced Hsp70 and constitutive Hsc70 chaperones, and also plays a role in maintaining their balance in the cell. When the chaperones carry no client proteins, CHIP catalyzes their polyubiquitination and subsequent proteasomal degradation. Although Hsp70 and Hsc70 are highly homologous in sequence and similar in structure, CHIP mediated ubiquitination promotes degradation of Hsp70 with a higher efficiency than for Hsc70. Here we report a detailed and systematic investigation to characterize if there are significant differences in the CHIP in vitro ubiquitination of human Hsp70 and Hsc70. Proteomic analysis by mass spectrometry revealed that only 12 of 39 detectable lysine residues were ubiquitinated by UbcH5a in Hsp70 and only 16 of 45 in Hsc70. The only conserved lysine identified as ubiquitinated in one but not the other heat shock protein was K159 in Hsc70. Ubiquitination assays with K-R ubiquitin mutants showed that multiple Ub chain types are formed and that the distribution is different for Hsp70 versus Hsc70. CHIP ubiquitination with the E2 enzyme Ube2W is predominantly directed to the N-terminal amine of the substrate; however, some internal lysine modifications were also detected. Together, our results provide a detailed view of the differences in CHIP ubiquitination of these two very similar proteins, and show a clear example where substantial differences in ubiquitination can be generated by a single E3 ligase in response to not only different E2 enzymes but subtle differences in the substrate.

## Introduction

The cellular response to stress induces expression of chaperone proteins such as heat shock protein 70 (Hsp70). These chaperones help to refold proteins and also work with ubiquitination machinery to degrade proteins that cannot be refolded [1]. When the crisis is over, the induced proteins must then be degraded to restore the normal cellular activities. Hsc70 is the constitutive chaperone homolog of Hsp70 that serves in a similar role to assist in protein refolding under normal cellular conditions. The significance of proper maintenance of the balance of constitutive and stress-induced chaperones such as Hsc70 and Hsp70 is underscored by alterations of the levels and clearance rate of pathogenic proteins like the neurodegeneration-associated tau when this balance is misregulated [2].

The heat shock protein co-chaperone CHIP functions with Hsp70 and Hsc70 in protein triage [3]. In addition to assisting in the refolding of substrates, CHIP is an E3 ubiquitin ligase that catalyzes the polyubiquitination of substrates, which leads to their degradation in the 26S proteasome. CHIP also plays a role in maintaining the cellular balance of Hsp70 and Hsc70 as it will polyubiquitinate these chaperones when they carry no client [4,5]. Studies in vitro and in cells have shown that whereas both Hsp70 and Hsc70 are ubiquitinated by CHIP, only the inducible form (Hsp70) is substantially degraded [6]. This is logical from a functional perspective as the inducible form must be removed when cell stress is alleviated whereas levels of the constitutive form need to remain constant. However, degradation of only Hsp70 is surprising because the two proteins share >86% sequence identity and 93% sequence conservation. This poses a significant challenge to our understanding of both the subtleties of polyubiquitin chain formation and the recognition of specific chain types as a signal for degradation.

Ubiquitination of proteins involves the action of a cascade of E1 activating, E2 conjugating and E3 ligating enzymes. Ubiquitination can signal for an array of different responses, which are determined by the extent (number) and type (chain linkage and forking of chains) of ubiquitin (Ub) molecules transferred to the substrate [7]. As with other post-translational modifications, the position of the modification on the substrate is a key, although not the sole, factor determining the functional outcome.

CHIP has been shown to produce all known types of ubiquitination modifications including K48-linked and forked Ub chains [8]. A previous study examined CHIP-mediated ubiquitination of Hsp70 and reported six sites that were ubiquitinated based on proteomic analysis with ~64% coverage of the protein [9]. Here, we report a more in-depth and systematic biochemical and proteomic analysis of CHIP ubiquitination of Hsp70 and also compare the results to the corresponding analysis of the highly similar Hsc70 protein. These studies represent one of the most in-depth investigations of in vitro ubiquitination reported to date and provide clear evidence of differences in the ubiquitination of these two homologous chaperones.

## Materials and Methods

### Protein purification

All proteins were purified from recombinant expression in *E*. *coli* and correspond to human sequences for each protein except for E1, which is the wheat Uba1. E1 (Uba1), UbcH5a, Ube2W, UbcH13, Uev1a, CHIP, Hsp70, and WT Ub were purified as before except that for some experiments the affinity tag of Hsp70 was not cleaved [10]. The plasmid expressing human Hsc70 was a gift from Jason Young (McGill University), and His-Hsc70 was purified using Ni-affinity and size exclusion chromatography. Plasmids encoding Ub mutants K0, K6R, K48R and K63R were a gift from Rachel Klevit (University of Washington) and His-Ub K11R was a gift from Michael Rape (University of California, Berkeley). The untagged Ub mutants were purified by lysing cells in buffer containing 30 mM ammonium acetate pH 5.1, then treating the cell lysate with acetic acid to lower the pH 4.8 and centrifuging to remove the precipitate. The protein was then further purified using cation exchange with ammonium acetate buffer and then size exclusion chromatography. All proteins were stored at -80°C in buffer containing 20 mM Tris pH 7.5, 1 mM DTT and either 50 mM NaCl (E1 and Ub) or 100 mM NaCl (E2s, CHIP, Hsc70, and Hsp70).

### In Vitro Ubiquitination

CHIP autoubiquitination was performed as previously described with the time of incubation for each sample as noted [10]. Substrate ubiquitination for Hsc70 and Hsp70 was optimized for a minimum concentration of CHIP to reduce auto-ubiquitination species and performed in a manner similar to the autoubiquitination reactions with 1.5 μM Hsc70 or Hsp70, 0.15 μM CHIP, and 40 μM Ub. Briefly, all reactions were incubated in buffer containing 100 mM NaCl, 40 mM Tris pH 7.5, 5 mM MgCl_2_, and 5 mM ATP at 30°C for the times indicated. The reaction products were then separated by SDS-PAGE with 4–12% gradient gels and either stained with Coomassie Blue or transferred to PVDF membrane for a western blot. Immunoblotting used either 1:3000 monoclonal mouse anti-Ub (Abcam), 1:3000 polyclonal rabbit anti-CHIP (Calbiochem), 1:2000 monoclonal mouse anti-Hsp70 (Enzo Life Sciences), or 1:3000 monoclonal mouse anti-GST (GenScript) and the appropriate HRP-fused secondary antibodies at 1:7500–10000.

### Proteasomal degradation

In vitro degradation was allowed to proceed in concert with ubiquitination. The reactions were altered to better match the previous work of Qian et al 2006 such that conditions included 100 nM E1, 2 μM E2, 3 μM CHIP, 1 μM Hsx70, 50 μM Ub, in 30 mM HEPES pH 7.5, 20 mM NaCl, 1 mM DTT, 5 mM MgCl_2_, 5 mM ATP with a creatine phosphate ATP-regeneration system. Reactions were incubated at 37°C for up to 2 hours with 45 nM 26S proteosome as indicated and the products analyzed as above for in vitro ubiquitination.

### Mass spectrometry proteomics

Following SDS-PAGE analyses, gels were stained with Coomassie stain, and Hsc70 and Hsp70 bands were excised and cut into 1 mm^3^ pieces. These gel pieces were first treated with 45 mM DTT for 20 minutes, followed by treatment with 100 mM iodoacetamide for 20 minutes. After destaining with 50% MeCN in 50 mM ammonium bicarbonate, the gel bands were digested with sequencing-grade trypsin in 25 mM ammonium bicarbonate overnight at 37°C. Peptides were extracted by gel dehydration (60% MeCN, 0.1% TFA), the extracts were dried by vacuum centrifugation, and peptides were reconstituted in 0.1% formic acid. Extracted peptides were then separated by reverse phase liquid chromatography and analyzed by tandem mass spectrometry (MS/MS). For LC-MS/MS analysis, the peptide extracts were first loaded onto a capillary reverse phase analytical column (360 μm O.D. x 100 μm I.D.) using an Eksigent NanoLC Ultra HPLC and autosampler. The analytical column was packed with 20 cm of C18 reverse phase material (Jupiter, 3 μm beads, 300 Å, Phenomenox), directly into a laser-pulled emitter tip. Peptides were gradient-eluted at a flow rate of 500 nL/min, and the mobile phase solvents consisted of 0.1% formic acid, 99.9% water (solvent A) and 0.1% formic acid, 99.9% acetonitrile (solvent B). A 90-minute gradient was performed, consisting of the following: 0–10 min, 2% B; 10–50 min, 2–40% B; 50–60 min, 40–95% B; 60–65 min, 95% B; 65–70 min 95–2% B; 70–90 min, 2% B.

Eluting peptides were mass analyzed on an LTQ Orbitrap XL or an LTQ Orbitrap Velos mass spectrometer (Thermo Scientific), each equipped with a nanoelectrospray ionization source. For the majority of LC-MS/MS analyses, the instruments were operated using a data-dependent method with dynamic exclusion enabled. Full scan (m/z 300–2000 or 400–2000) spectra were acquired with the Orbitrap (resolution 60,000). For LTQ Orbitrap XL analyses, the top 5 most abundant ions in each MS scan were selected for fragmentation via collision-induced dissociation (CID) in the LTQ ion trap. A data-dependent method involving selection of the top 16 most abundant ions per MS scan was used for LTQ Orbitrap Velos analyses. An isolation width of 2 m/z, activation time of 10 or 30 ms, and 35% normalized collision energy were used to generate MS2 spectra. Dynamic exclusion settings allowed for a repeat count of 2 within a repeat duration of 10 sec, and the exclusion duration time was set to 15 sec. For selected LC-MS/MS analyses, the LTQ Orbitrap Velos was operated using a method consisting of data-dependent and targeted scan events, for which specific *m/z* values corresponding to Hsp70 or Hsc70 ubiquitinated peptides of interest were provided in the data acquisition method to facilitate targeted MS/MS spectra despite the intensity of peptide precursors.

For identification of Hsp70 and Hsc70 peptides, tandem mass spectra were searched with Sequest [11] against an *E*. *coli* subset database created from the UniprotKB protein database (www.uniprot.org), appended with protein sequences for the recombinantly expressed proteins E1, UbcH5a, Ube2W, UbcH13, Uev1a, CHIP, Hsc70, Hsp70 and Ub depending on their presence in the sample. Variable modifications of +57.0214 on Cys (carbamidomethylation), +15.9949 on Met (oxidation), and +114.0429 on Lys residues (corresponding to the diGly remnant that remains after tryptic cleavage of ubiquitin) were included for database searching. Search results were assembled using Scaffold 3.6.4 (Proteome Software). Spectra acquired of Hsc70 and Hsp70 peptides were then inspected using Xcalibur 2.1 Qual Browser software (Thermo Scientific). Tandem mass spectra of ubiquitinated peptides as well as spectra acquired of the corresponding unmodified peptide forms were examined by manual interrogation, and sites of ubiquitination on modified peptides were validated. Structural models were created based on the ADP-bound form of the *E*. *coli* homolog DnaK (PDBID 2KHO[12]) using the SWISS-MODEL web server[13].

## Results

The objective of this study was to systematically characterize the ubiquitination of Hsp70 by the E3 ubiquitin ligase CHIP and identify any differences relative to ubiquitination of Hsc70. Our laboratory previously established that CHIP could produce polyubiquitin chains only with either an E2 from the UbcH5 family or the combination of a mono-ubiquitinating E2 (Ube2W or UbcH6) with the chain-building E2 UbcH13/Uev1 [10]. Our studies focused on the analysis of UbcH5-mediated ubiquitination because it generates a diversity of products that could lead to differences in their rates of proteasomal degradation. Nevertheless, we also analyzed CHIP ubiquitination with the Ube2W E2 enzyme to determine if the modification of the heat shock proteins was the same as for CHIP ubiquitination with UbcH5.

Importantly, a link between in vitro results and in vivo (cellular) degradation has been made in the previous study of CHIP-mediated ubiquitination of Hsx70 proteins [6], albeit with a greater emphasis on degradation relative to our focus on ubiquitination. In particular, that study showed that Hsp70 was degraded faster and more completely than Hsc70 in both in vitro assays using purified components and in mouse fibroblast cells. While, our goal was to follow up on the initial observation and obtain detailed knowledge of CHIP-mediated ubiquitination of the Hsx70 proteins, to ensure the two studies were aligned, we confirmed that the ubiquitinated protein produced by our assays follows the same trend of degradation as reported previously (*vide infra*).

### Identification of ubiquitination sites by tandem mass spectrometry

**Fig 1:**
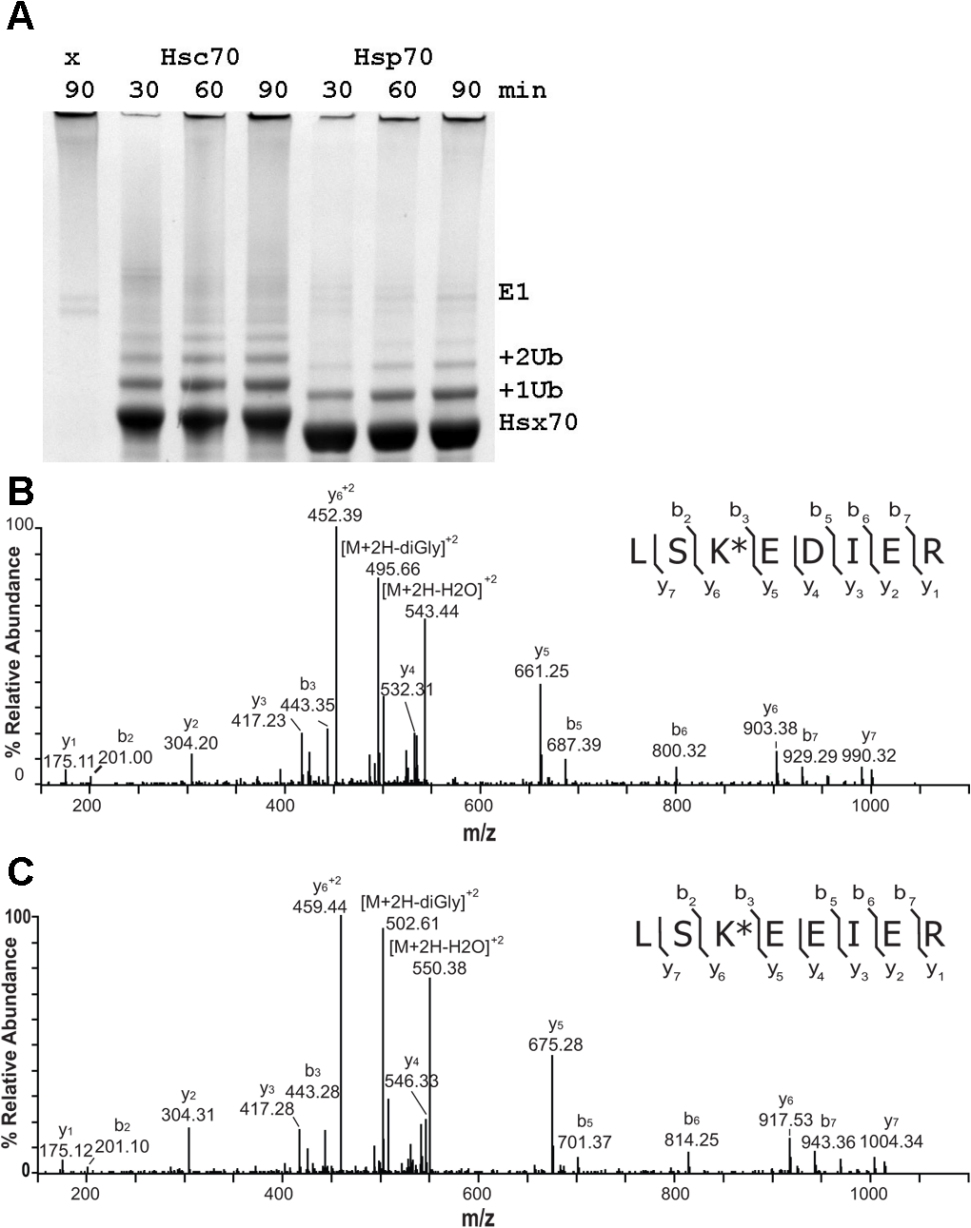
Identification of ubiquitinated lysines for Hsc70-Ub and Hsp70-Ub. A) In vitro ubiquitination reactions with CHIP and UbcH5a were incubated up to 90 minutes as labeled and the products were separated by SDS-PAGE. The singly and doubly-ubiquitinated bands were excised for in-gel digestion and LC-MS/MS analysis. B) Example MS/MS spectrum of Hsc70 LSK(GG)EDIER peptide identifying that Ub was attached to K512. The K* indicates the lysine residue is modified by the addition of a di-glycine peptide. Observed b- and y-type product ions, resulting from amide bond cleavage following collision-induced dissociation, are annotated above the corresponding product ion peaks in the spectrum. C) Example MS/MS spectrum of Hsp70 peptide LSK(GG)EEIER for the equivalent site.

**Fig 2:**
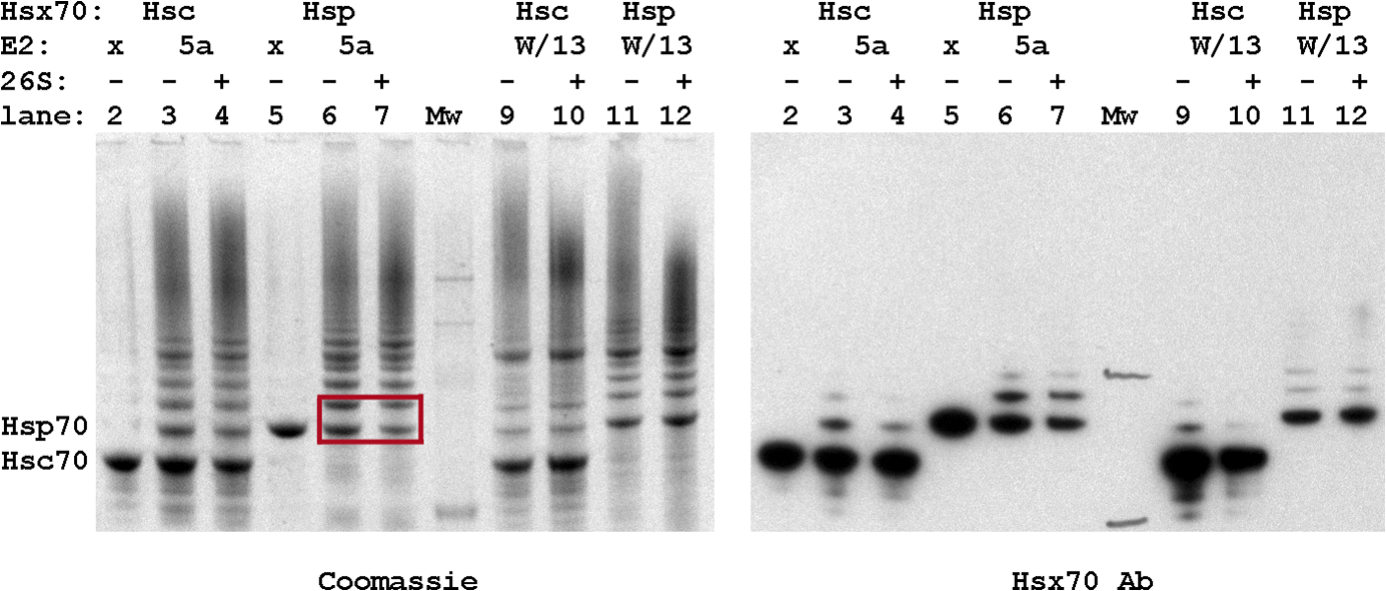
Hsp70 is more rapidly degraded than Hsc70 by 26S in vitro. Concurrent ubiquitination and degradation reactions were incubated for 2 hours at 37°C before separating the products on the gel. Key bands are highlighted and quantification of both the Coomassie stained and immunoblot analyses confirm that only Hsp70 ubiquitinated by UbcH5a is significantly degraded under these conditions.

Hsp70 and Hsc70 have a high degree of sequence identity (86%) and most lysines are conserved (S1 Fig). While there is one reported analysis of human Hsp70 ubiquitination by CHIP with UbcH5, no data are available for Hsc70. We therefore performed a comparative proteomic analysis to extend the analysis of CHIP ubiquitination of Hsp70 and identify the differences with respect to ubiquitination of Hsc70. The in vitro ubiquitination reactions were optimized to obtain the highest yields and resolution of the ubiquitination ladder products under multiple turnover conditions (Fig 1A). To support the correlation of the in vitro data with the degradation in vivo, as has been established previously, we examined the degradation of ubiquitinated Hsp70 relative to ubiquitinated Hsc70 in experiments with purified 26S proteasome added (Fig 2). While these data do not exactly replicate the previously reported results, they show that the CHIP-ubiquitinated proteins produced in our assays exhibit the same trend of preferential degradation of Hsp70 versus Hsc70 in vitro and in vivo [6]. Thus, the in vitro ubiquitinated proteins are distinguishable in some manner, which is investigated below.

Our analysis focused on the +1 Ub and +2 Ub bands, which were excised from the gel and trypsin digested, then the resultant peptides were extracted for LC-MS/MS analysis. Trypsin cleavage of a Ub modified lysine leaves a signature diGly tag that can be recognized as a post-translational modification of +114 Da. The samples from the +1 Ub bands are expected to contain a mixture of many different mono-ubiquitinated species representing the range of ubiquitination sites that are produced, while the +2 Ub bands will contain molecules with either two separate ubiquitination sites or a di-Ub addition to a single site. Examples of tandem mass spectra of ubiquitinated Hsx70 (both Hsp70 and Hsc70) peptides are shown in Fig 1B and 1C.

Optimization of the proteomic analysis resulted in identification of peptides representing 84% of the sequence of Hsp70 compared to the 64% coverage in the previous study (Table 1, S1 Fig). This enabled 39 of the 50 lysines in Hsp70 to be monitored for modification. Most of the lysine residues that could not be analyzed are located in regions of the proteins that when trypsin digested result in very short peptides, and therefore are not detectable in our LC-MS/MS experiments. Remarkably, while we anticipated that ubiquitination would be quite promiscuous, only 12 lysines in Hsp70 were modified at detectable levels. This included all 6 sites previously reported for Hsp70 [9] and an additional 6 sites in the new regions of the protein covered by our analysis. The di-ubiquitinated Hsp70 sample was specifically analyzed to look for Ub-Ub bonds and only K48-linked chains were found. This is in contrast to the four types of Ub linkages reported previously [9]. While the mass spectrometry experiments are not quantitative, predominance of K48-linked ubiquitin chains on Hsp70 would be consistent with degradation of the ubiquitinated protein.

**Table 1:** Summary of results from mass spectrometry proteomics analysis of +1 or +2 Ub samples from Fig 1A.

	Sequence coverage	Lys observed	Ub sites
Hsc70	89%	45/54	16
Hsp70	84%	39/50	12
Ub (from Hsc)	92%	6/7	3
Ub (from Hsp)	92%	6/7	1 (K48)
CHIP	40%	7/20	1 (K22)

Proteomic analysis of Hsc70 was also completed with 89% of the sequence observed. For this protein 45 of the 54 lysines could be monitored, but again only a subset was detected with Ub modification. These 16 sites include 12 sites that are either identical to or within three residues of the Hsp70 ubiquitination sites (S1 Fig). Among the four lysine residues uniquely ubiquitinated in Hsc70, three involved sites for which there is no analogous lysine in Hsp70 (K458, K531, and K601). The fourth site, K159, is a lysine in Hsp70 and therefore represents the only direct difference in the ubiquitination sites for the Hsx70 proteins. To validate that this site is not ubiquitinated in Hsp70, a tandem mass spectrometry experiment was performed to target the predicted peptide ion of the K159-ubiquitinated Hsp70 peptide. Only a very weak signal could be observed for the expected mass, and the peptide could not be identified with confidence. Hence, if this site is modified at all it is at a very low level. The di-ubiquitinated Hsc70 was also specifically analyzed to look for Ub-Ub bonds and three types of linkages were observed for Hsc70 (K6, K11 and K48). It is important to note that these experiments are not quantitative in nature so firm conclusions cannot be drawn. Nevertheless, the results suggest that K48-linked Ub is the predominant product of ubiquitination of Hsp70 by CHIP, but not necessarily for Hsc70.

**Fig 3:**
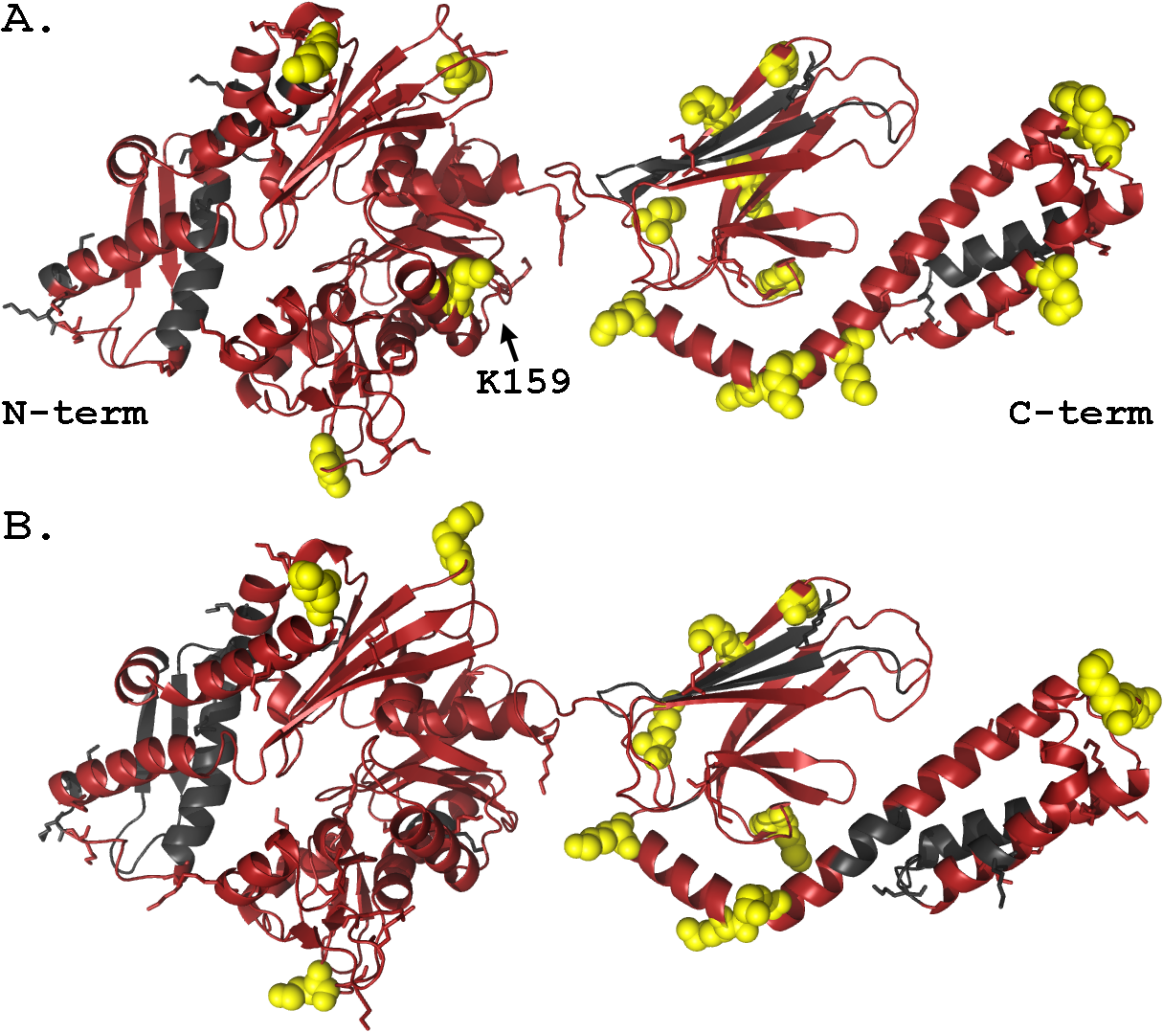
CHIP ubiquitination of Hsc70 (A) and Hsp70 (B) is wide-spread but largely focused on the C-terminal half of the protein. Homology models based on the structure of DnaK (PDB ID 2KHO) were created using SWISS-MODEL, and are here used to map the sequence coverage and ubiquitination sites found by mass spectrometry proteomics. Observed regions are colored in red, with unobserved regions in grey. All lysine side-chains are explicitly shown with confirmed ubiquitination sites highlighted in yellow.

The high overall coverage of the proteins in the proteomic analysis enabled us to investigate if a structural basis for the limited number of ubiquitination sites observed is evident. Mapping the ubiquitination results onto homology models of Hsc70 and Hsp70 reveals a broad distribution of ubiquitination sites with a slight concentration of modified sites in the C-terminal half of the proteins (Fig 3). This graphical analysis demonstrates that ubiquitination is not localized to a single face of either protein. CHIP binds to the dynamically disordered C-termini of Hsc70 and Hsp70 (not shown in Fig 3) and both Hsx70 proteins are known to be structurally dynamic with well-characterized conformational changes over the course of the ATP cycle. Hence, it is likely that a wide range of orientations of CHIP and the substrate are sampled during the assay, which should allow for ubiquitination of nearly any exposed Hsx70 lysine residue.

### Analysis of Hsx70 polyubiquitin linkage using Ub point mutations

To obtain further information on the Ub chain linkages formed on Hsc70 and Hsp70, ubiquitination reactions were performed using four single lysine to arginine Ub mutants (K6R, K11R, K48R, K63R) and Ub K0, which has all seven lysines mutated to arginine and cannot form lysine-linked chains. The loss of a single Lys site on Ub will restrict ubiquitination if that is the preferred site, but often will not entirely block activity as other sites can be used effectively under the in vitro conditions used. In order to perform studies of Hsp70 and Hsc70 ubiquitination, it is necessary to determine if the Ub mutants have a fundamental effect on CHIP activity even though all are readily conjugated to UbcH5a.

**Fig 4:**
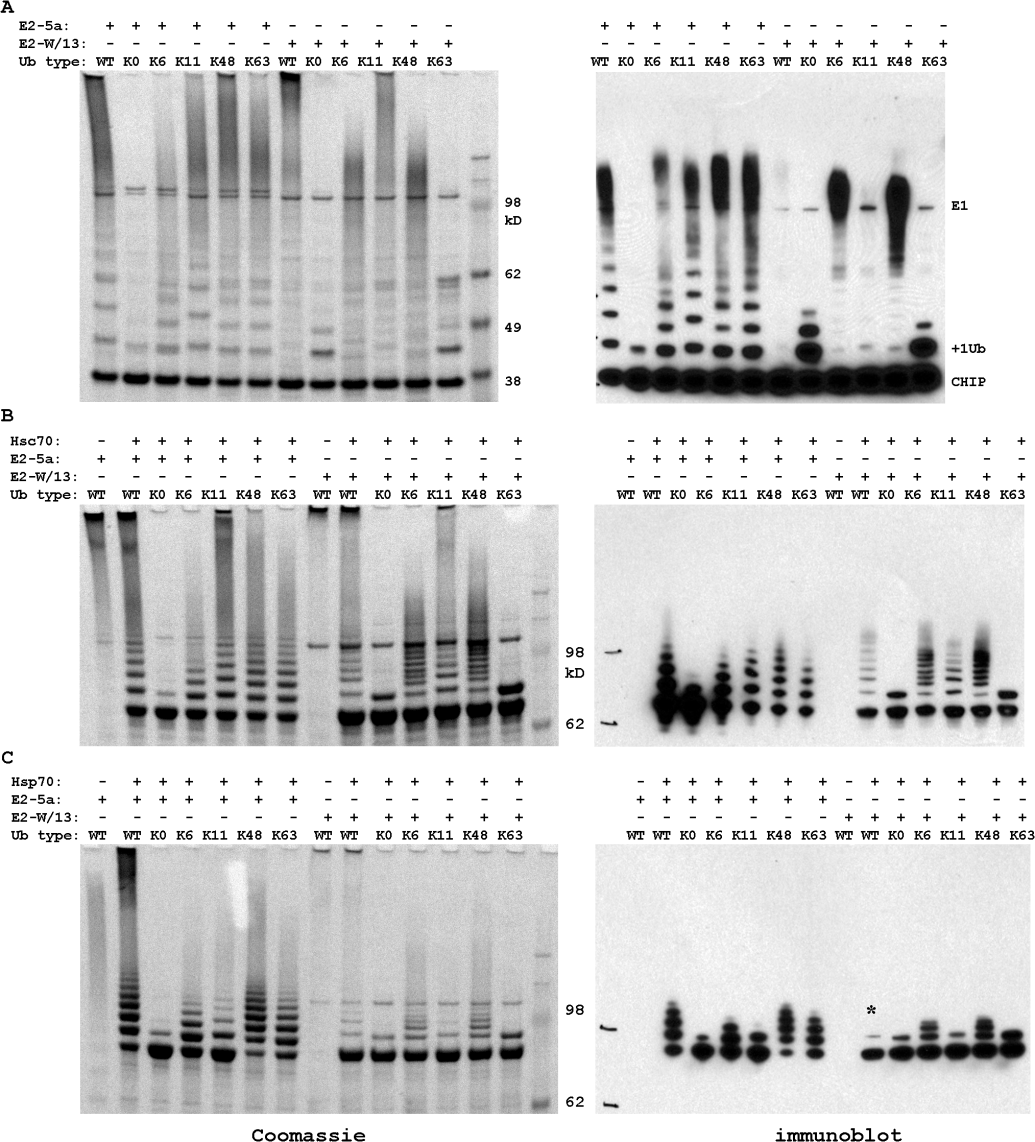
Ubiquitin mutants alter the rate of reaction in vitro. All Ub species were incubated for 30 minutes with UbcH5a or Ube2W+ UbcH13/Uev1a as the E2 as labeled. Both the Coomassie stain, left, and immunoblot, right, are shown for each set of reactions. A) CHIP autoubiquitination, B) Hsc70 ubiquitination by CHIP, and C) Hsp70 ubiquitination by CHIP. The * indicates a weak signal in the western blot that is more clearly seen by Coomassie stain. Note that K11R Ub retains a fusion tag that results in a larger protein and thus larger ladder spacing.

To this end we performed CHIP auto-ubiquitination assays, which we had characterized extensively for WT Ub in a previous study [10]. We note that CHIP exhibits very robust auto-ubiquitination activity resulting in an amount of polyubiquitinated CHIP molecules that can be readily identified by Western blotting. Fig 4A compares results from CHIP autoubiquitination reactions with WT Ub and each of the 5 mutants. K11R Ub is a fusion protein with an N-terminal His tag and as such is a slightly larger protein and produces wider spacing in the Ub ladder. As expected, K0 Ub greatly reduced ubiquitination and bands are observed for only one or two Ub molecules added. The +2 Ub band presumably arises from addition to two different sites on the substrate. The products of Ub K11R, K48R and K63R reactions are all similar to WT Ub. The only mutant that did not follow expectations was K6R, for which the reactions seemed to produce less ubiquitinated product overall, particularly in the amount of highly ubiquitinated species. Together, these results provide the essential background for analyzing the effect of Ub mutations on CHIP substrate ubiquitination.

CHIP auto-ubiquitination is very robust. In order to reduce the background in substrate ubiquitination reactions, the concentration of CHIP was reduced to the catalytic minimum. Despite this precaution, Western blots with substrate, CHIP and Ub antibodies of Hsx70 reactions reveal that most if not all of the high molecular weight species observed in Coomassie stained gels correspond to polyubiquitinated CHIP molecules (S2 Fig). Hence, we find that ubiquitination of the Hsx70 substrates by CHIP in vitro results in the addition of only a few (1–4) Ub molecules and little if any evidence of extensive polyubiquitination when the E3 is at catalytic concentrations.

Hsc70 ubiquitination by CHIP appears to respond to Ub mutations in the same way as CHIP autoubiquitination. As expected, the Hsc70 ubiquitination assays show K0 Ub dramatically reduces the extent of substrate ubiquitination compared to WT Ub (Fig 4B). The proteomics based identification of Ub chain linkages predicts that Hsc70 ubiquitination with CHIP and UbcH5a, for which K6, K11, and K48 chains were observed, should be perturbed by the corresponding Ub K-R mutants. However, of these three Ub mutants only K6R appears to perturb the reaction, and this may be due to the reduction in CHIP activity with K6R Ub. In addition, we were surprised to observe that the K63R Ub mutant perturbed Hsc70 ubiquitination because we had not observed K63-linked chains in the proteomics study.

Hsp70 ubiquitination showed much greater variation with the Ub mutants (Fig 4C). Again, K0 Ub allows only monoubiquitination of Hsp70. The proteomics based identification of Ub chain linkages predicts that Hsp70 ubiquitination should be perturbed by the Ub K48R mutant; however, K48R Ub does not appear to alter the extent of ubiquitination while K11R Ub does. Moreover, the reactions for both K6R and K63R Ub were also perturbed, albeit to a much lesser extent than K11R.

To obtain further insight, the assays with Ub mutants were repeated with the addition of26S proteasome using the standard approach of monitoring the amount of intact, unmodified substrate (S3 Fig). Importantly, although only coarse estimates of degradation can be made, as noted above for WT Ub more robust degradation is observed for Hsp70 than Hsc70. As a consequence, we find little if any variability in the degradation of Hsc70 with the different mutants. In contrast, differences in the extent of degradation of Hsp70 for the different Ub mutants are detected. Confidence in the analysis is raised because the results appear to parallel the effect of Ub mutants in the assays where total ubiquitination is monitored (Fig 4B and 4C). Consistent with the very small amount of substrate ubiquitination observed in the absence of proteasome, the results for K0 and K6R Ub showed little evidence of degradation. For the K11R, K48R, and K63R Ub mutants, ubiquitination activity similar to WT was observed and degradation of Hsp70 for these mutants was similar to WT.

### Hsx70 ubiquitination with the Ube2W and UbcH13/Uev1 E2 conjugating enzymes

Combined with our results from UbcH5a ubiquitination we investigated if the substrate specificity of CHIP depends on the identity of the E2. Moreover, analysis of ubiquitination by the combination of Ube2W and UbcH13/Uev1 E2 conjugating enzymes (W/13) provides further insight into ubiquitination by CHIP because it separates the initial mono-ubiquitination of substrates by Ube2W from polyubiquitination through K63-linked chains by UbcH13/Uev1.

We first examined CHIP autoubiquitination for W/13 and compared the results to the products from reactions using UbcH5a (Fig 4A). As expected, since the W/13 E2 combination produces K63 chains, use of K0 or K63R Ub results in substantially reduced ubiquitination products and K11R reactions were similar to the WT control. Surprisingly, both K6R and K48R exhibited a marked decrease in the extent of ubiquitination such that no very high molecular weight species were produced. One possible explanation for these observations is that critical non-covalent interactions of Ub with Uev1a require the surface including K6 and K48, and that without them the chain extension activity of UbcH13/Uev1a is diminished[14].

We next turned to analysis of CHIP ubiquitination of Hsc70 with W/13 (Fig 4B). WT Ub produces a faint Ub ladder. This is reduced to monoubiquitination with both K0 and K63R Ub as neither can produce chains with W/13. The other three Ub mutants all have a ladder of products that resemble the extent of ubiquitination with WT Ub. In contrast, the ubiquitination reactions for Hsp70 with W/13 have much lower levels of ubiquitination overall, with only +1 and +2 Ub bands prominent even for WT Ub. As expected, K0 and K63R Ub are only capable of monoubiquitination, and K6R and K48R Ub products are similar to WT Ub. K11R Ub generates much less reaction, as was observed with the E2 enzyme UbcH5a, which suggests a general role of K11 in CHIP ubiquitination of Hsp70.

A surprising observation was made in the Hsx70 ubiquitination reactions with the E2 enzymes W/13 and the K6R and K48R Ub mutants. The Ub ladders produced from these reactions included not only the normal ladder of +1, +2, +3 Ub but also produced extra bands in between the expected bands. To obtain further insight, we examined reactions with Ube2W alone to isolate the monoubiquitination function (S4 Fig). Initial mass spectrometry proteomics analysis of the band at the +1.5 Ub region on the gel from the reaction with Hsp70, W/13, and K48R Ub (S4 Fig, lane 13) did not identify any lysine ubiquitination sites on Hsp70 despite good coverage by mass spectrometry (S5 Fig).

Previous studies have strongly implied that Ube2W is only capable of catalyzing monoubiquitination [10,15]. Moreover, recent reports have shown that Ube2W will predominantly attach Ub to the N-terminus of substrates[16,17]. Indeed, when searches were limited to ubiquitination of lysine residues, no modifications were observed. After extending the search, the proteomic analysis of Hsp70-Ub bands produced with Ube2W and WT, K0 and K48R Ub (bands from S5 Fig lanes 3, 4, and 7), all showed Hsp70 is predominantly modified on the N-terminus. In fact, the signal for this linear peptide is approximately 100-fold stronger than other ubiquitinated peptides we have seen and the signature diGly addition can be clearly identified in the y-ion series (S6 Fig).

In addition to the primary site N-terminal ubiquitination with Ube2W, experiments with WT Ub revealed two internal Hsp70 lysines were ubiquitinated, K507 and K524. These signals were weak and required targeted analysis to obtain sufficient signal intensity to confirm the peptide assignment. Although not quantitative, these observations imply the modifications occur at much lower levels than the N-terminal modification. Importantly, the secondary ubiquitination sites provide an explanation for the +2, etc bands in reactions with WT and K11R Ub since there are multiple acceptor sites. The single +1 Ub band with the other Ub mutants (e.g. K0) and lack of identifiable internal lysine sites by proteomics both suggest there is less internal ubiquitination in reactions with these mutants.

**Fig 5:**
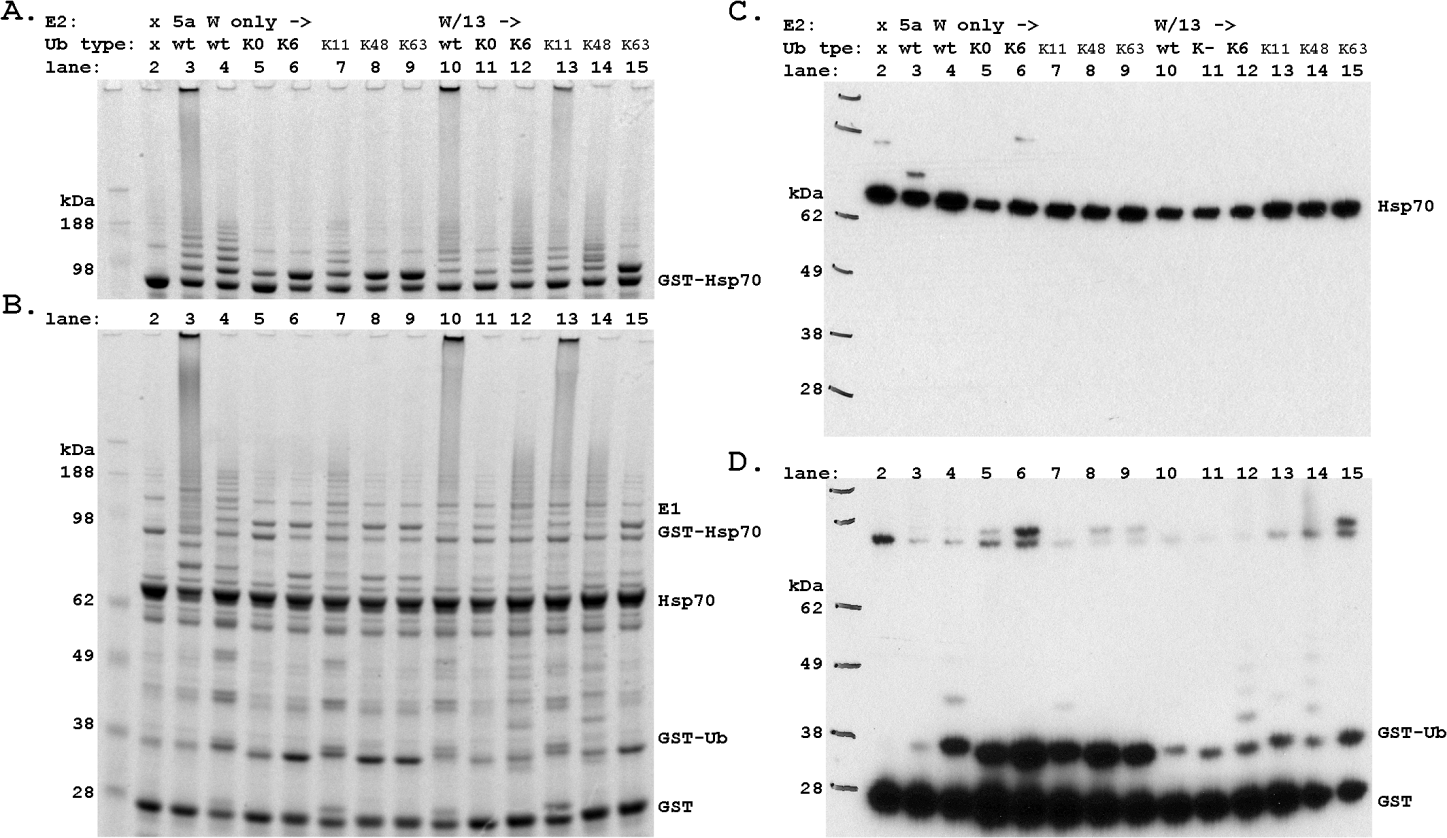
Ubiquitin is predominantly attached to the N-terminus of His-GST-Hsp70 by Ube2W. In vitro ubiquitination reactions with the fusion protein and various Ub mutants were incubated as above for 30 min (A). To isolate N-terminal ubiquitination, 0.2 μM H3C protease (to cleave His-GST from Hsp70) and 50 mM L-Cys (to quench E1 activity) were then added to a portion of the reaction and allowed to incubate for 30 min at room temperature prior to separation by SDS-PAGE (B). The cleaved reaction products in B were also investigated by western blots for C) Hsp70 and D) GST. The majority of the ubiquitination products in all reactions containing Ube2W (lanes 4–15) follow His-GST and indicate ubiquitination of the N-terminus.

To further investigate the products of Ube2W reactions and determine the origin of the +1.5 Ub bands, we performed experiments using the method of Tatham et al.[16] in which the Hsp70 substrate is fused to a cleavable tag to enable N-terminal ubiquitination to be distinguished from internal lysine ubiquitination of the substrate. Ubiquitination products from reactions with the His-GST-Hsp70 fusion protein were treated to cleave the fusion protein, then all products were separated by SDS-PAGE (Fig 5A and 5B). Hsx70 and GST antibodies were used to confirm the identity of cleaved fragments (Fig 5C and 5D). As expected, the majority of the ubiquitinated species for all reactions containing Ube2W were associated with the N-terminal His-GST fragment with little ubiquitination of Hsp70 itself. In reactions with UbcH5a, some Hsp70-Ub was detected confirming the observation of internal lysine ubiquitination (Fig 5C; lane 3). Hsp70-Ub was also observed in reactions with Ube2W and WT Ub albeit at much lower levels (Fig 5B; lane 4).

These results prompted a further investigation of the origin of the +1.5 Ub band from the K48R Ub reaction with W/13 (S4 Fig, lane 13). To extend the proteomics analysis, a targeted search was performed for the internal lysine sites previously found with Ube2W and WT Ub (K507 and K524), but only the N-terminal ubiquitination could be confirmed. Interestingly, this analysis revealed the presence of K63-linked di-Ub, which indicates that at least some of the species in this band have linked Ub molecules.

In the more sensitive biochemical experiments with the cleavable His-GST-Hsp70 fusion protein, the +1.5 Ub intermediate bands are seen for K6R and K48R Ub in W/13 reactions, and following cleavage of the protein the +1.5 Ub ubiquitination maps to the N-terminal GST fragment rather than the Hsp70 protein (Fig 5D; lanes 12, 14). Combined with the evidence for K63-linked di-Ub in the proteomics analysis, we propose that the +1.5 band comes from Hsp70-Ub_(2)_ where the first Ub is attached to the N-terminus of the substrate and the second Ub is attached to K63 of the first. Moreover, the other +n.5 bands would represent additional K63-linked extensions of the Ub chain. The aberrant mobility of this species in denaturing PAGE analysis is thus attributed to the forked di-ubiquitin at the N-terminus, which likely causes the protein to move faster through the gel matrix than linear peptides of the same mass.

## Discussion

Our analysis of CHIP ubiquitination products greatly extends knowledge of the activity of this E3 ligase and its specificity towards substrates. Previous studies found only six ubiquitination sites on Hsp70 [9], but with the significantly greater coverage in the proteomic analysis we find there are 6 additional sites that are modified. In addition, we report 16 sites are modified on the highly homologous Hsc70. Interestingly, the sites for both Hsc70 and Hsp70 are distributed throughout and on multiple faces of the proteins, and the sites are nearly identical between the two highly conserved proteins. This pattern of Ub modification implies that there are multiple orientations for Ub to transfer to these substrates. Since the ubiquitination machinery has multiple flexible linkers (including the E2-Ub bond, the CHIP dimer, and the Hsx70 C-terminus) it is expected that most of the surface exposed lysines would be available for ubiquitination [18–20]. However, only 12 of the 39 observed Hsp70 lysines and 16 of the 45 observed Hsc70 lysines are modified. As we observe good coverage and strong mass spectrometry signals for the unmodified protein, we can infer that most sites are not ubiquitinated at significant levels. Hence, modification does not occur for every accessible site. This implies that the selection of sites of ubiquitination involves multiple factors, not just accessibility to the ubiquitination machinery.

Ubiquitin linkage types also reflect the specificity of the ubiquitination machinery. Previous studies have reported that CHIP can produce all possible Ub linkages and that Hsp70 is modified in vitro with K6, K11, K48, and K63-linked chains [8,9]. Although we also observe diversity in linkage types while noting K48 chains are clearly dominant for Hsp70. This is significantly different from Hsc70, for which the majority of Hsc70 modifications are spread between K6, K11 and K48. In a previous report on Hsc70 ubiquitination in Cos-7 cells [21], single lysine mutations K29R and K48R had no apparent effect on ubiquitination, fully consistent with our observations. However, no effect was observed for the K63R mutant in that study, whereas we found a reduction in overall ubiquitination activity. We attribute this difference to the greater sensitivity of our assays to a reduction in the rate of ubiquitination. In contrast, their observation that the Ub double mutant K29R, K63R blocks all but mono-ubiquitination of Hsc70 is not supported by our data. It is possible that this combination of Ub mutations significantly inhibits E2 or E3 function, but importantly, there are substantial differences in the experiment conditions between our studies and the previous report. In this report, by performing experiments under identical conditions, there is very high confidence in the intrinsic differences we find between the predominance of K48 chains from CHIP-mediated ubiquitination of Hsp70 versus the distribution of K6, K11, and K48 chains for Hsc70.

The analysis of Ub chain types by proteomics methods versus the effects of ubiquitin mutants on ubiquitination reactions proved highly insightful, largely because these two methods are orthogonal and provide complementary views. Consider the case of Hsp70, for which one might anticipate a considerable change in the extent of ubiquitination of Hsp70 in reactions with the K48R Ub mutant. The fact that the no significant difference in ubiquitination is observed seems to suggest that the proteomic and biochemical approaches do not agree; however, the apparent discrepancy can be explained by the generation of chains on the other lysine sites on ubiquitin when the preferred K48 option is not available. This phenomenon also greatly complicates and effectively precludes detailed interpretation of results obtained in degradation assays with Ub K-R mutants. In our experiments, the extent of degradation of Hsp70 was sufficient to discern differences between the mutants, but this is not the case for Hsc70. The K-R mutation of any single lysine is insufficient to greatly alter degradation of Hsc70 because only a limited portion of the chains that are most effectively recognized and processed by the proteasome are formed with WT Ub and any of the mutants. In contrast, the reduction in degradation of Hsp70 observed for K48R is consistent with the predominance of K48 chains for this substrate and more effective degradation of Hsp70 relative to Hsc70.

Mass spectrometry-based proteomics is limited by the fact that the observation of a peptide is highly dependent on multiple factors, including its mass, charge, ionization ability, hydrophilicity, fragmentation characteristics etc. [22,23]. Moreover, specialized approaches are required to obtain quantitative data and are of limited value for a broad survey such as of the large Hsc70 and Hsp70 proteins. The biochemical assays with Ub mutants are complementary because they provide an overview of the total ubiquitination modification of the substrate; however, these data do not distinguish the different chain types that are present. Hence, it is analysis of the ensemble of data that provides the greatest insight.

One concern about Hsx70 ubiquitination by CHIP is that no chains containing >4 Ub molecules were observed and therefore the ubiquitination reaction may not be processive. This is reflected in the observation of high molecular weight poly-Ub CHIP species, yet no poly-Ub Hsx70 for the same samples (see S2 Fig). A similar observation was reported in a previous study of Hsp70 ubiquitination and degradation, which found a correlation between in vitro and in vivo studies [6]. Polyubiquitinated Hsx70 has been observed by western blot in other studies but only under conditions with high concentrations of CHIP and where the Ub ladder is not resolved [24]. While Ub could interfere with antibody recognition of Hsc70-Ub_(n)_, our controls suggest that the high molecular weight species identified by Coomassie stain in our assays are unaffected by the presence of substrate and thus are primarily ubiquitinated CHIP.

The length of Ub chains has important implications for substrate recognition and recruitment to the 26S proteasome [25]. For proteins with a well-structured domain, ubiquitination and subsequent recognition by the 19S activator of the proteasome is required. It is commonly believed that a chain of at least four ubiquitin molecules is required for recognition by the 19S activator. Degradation of ubiquitinated substrates with fewer Ub has been reported when the ubiquitination site is adjacent to a significantly unstructured region [26]. In fact, mono-ubiquitination may be sufficient for recruitment to the proteasome and several examples have appeared in the literature in the last few years [27].

What allows substrates with fewer than four-Ub chains to be degraded in the proteasome? The main benefit of a longer Ub chain is that it allows simultaneous interaction with multiple ubiquitin interaction motifs and consequently will bind tighter to 19S activator proteins. Although a short chain may not bind as strongly, the adjacent unstructured regions of proteins could lower the energy barrier to pull the substrate into the core of the 19S activator. Hsp70 and Hsc70 contain three folded domains with an unstructured C-terminal tail and multiple flexible hinges that facilitate conformational exchange during the ATPase cycle. Additionally, the largest sequence divergence in the two proteins occurs at the C-terminus where Hsp70 has five more residues contributing to the flexible region. Thus, flexibility at the C-terminus of Hsp70 and Hsc70 may not only provide a mechanism for substrate degradation with the addition of only 1–4 Ub molecules upon CHIP ubiquitination, but may also contribute to the differential degradation of these two homologous proteins.

The differences we find in CHIP ubiquitination of Hsp70 versus Hsc70 may provide clues for why these two chaperones are degraded at much different rates in the proteasome. Although data that directly show a strong correlation between chain type and degradation by the 26S proteasome is lacking, one can speculate on possible explanations, including one or more of the following factors: (i) subtle differences in which lysine residues on the two proteins are modified; (ii) which Ub-Ub linkage is dominant; (iii) the flexibility of the protein. Also, one cannot discount a key role for one or more lysine residues that are not detected in the proteomic analysis. Most importantly, in extrapolating to the cellular context, additional accessory factors and the role of de-ubiquitinases need to be considered. It is clear that investigations of all of these factors needs to be undertaken to discern how it is possible that the level of these chaperones with such high homology and structural similarity can be appropriately balanced to support protein triage both in cellular homeostasis and during stress response.

## Conclusion

The detailed analysis of ubiquitinated Hsx70 proteins presented here refines knowledge of the ubiquitination activity of CHIP and extends the earlier experiments that first examined CHIP mediated ubiquitination. Our proteomic and in vitro ubiquitination analyses indicate that the CHIP ubiquitination machinery generates a range of ubiquitination modifications of both chaperones. However, within this context specific differences in the ubiquitination of Hsp70 and Hsc70 occur, even though they are highly homologous and structurally similar proteins. Importantly, our results represent one of only very few in-depth comparative studies demonstrating that two highly homologous proteins can be differentially ubiquitinated. In addition, our studies of reactions with Ub mutants confirm an underlying plasticity in the ubiquitination machinery that can compensate for the blockage of certain chain linkages and even alter chain type specificity. Ubiquitinated Hsp70 has modifications spread throughout the protein and primarily K48-linked Ub chains, whereas Hsc70 has a wider range of sites of ubiquitination and of types of Ub chains formed. The larger amount of K48 chains on Hsp70 is intriguing, if one assumes that these lead to higher efficiency of substrate recognition and/or degradation by the 26S proteasome. Additional data on the correlation between the distribution of Ub chain types and degradation would be of great interest to confirm or refute this hypothesis. In summary, our data have provided new insights into the specificity of the CHIP E3 ligase and the sensitivity of ubiquitination machinery to even subtle differences in the sequence and structure of its substrates.

## Supporting Information

S1 Fig Residue-level details of the proteomics results for the studies with UbcH5a and CHIP with both Hsc70 and Hsp70. (DOCX)

S2 Fig High molecular weight ubiquitination products are mostly CHIP-Ub_(n)_. In vitro ubiquitination reactions of 30 minutes without and with substrates were blotted for Hsx70, Ub and CHIP with the respective antibodies as labeled. While high molecular weight species are identified as containing CHIP and Ub, only products containing +1–2 Ub can be observed for the Hsx70 substrates.
(TIF)

S3 Fig Ub mutants alter the rate of Hsx70 degradation in vitro. In the same manner as Fig 2, concurrent ubiquitination and degradation reactions with UbcH5a and the Ub type indicated were incubated for 2 hours at 37°C. The amount of unmodified Hsx70 was quantified and the relative amount of protein remaining in each reaction is indicated.
(TIF)

S4 Fig Ube2W has a unique ubiquitination pattern for substrates of CHIP. Hsp70 ubiquitination reactions were incubated for 30 minutes as above except using Ube2W alone or in combination with Ubc13/Uev1a the E2. Samples for mass spectrometry proteomics were taken from lanes 3, 4, 7, and 13 as noted in the text.
(TIF)

S5 Fig Residue-level details of the proteomics results for the studies with Ube2W and CHIP with Hsp70. (DOCX)

S6 Fig Confirmation of N-terminal ubiquitination of Hsp70 by Ube2W. The annotated MS/MS spectrum for the peptide (gg)-GPGSMAK identifies the signature Ub diGly tag attached directly to the N-terminus of the Hsp70 protein.
(TIF)
