# Supplementary material for: Biochemical and Proteomic Analysis of Ubiquitination of Hsc70 and Hsp70 by the E3 Ligase CHIP
Source: PLoS One. 2015 May 26;10(5):e0128240. doi: 10.1371/journal.pone.0128240 (PMC4444009; doi:10.1371/journal.pone.0128240)
Supplement: S1 Fig — (DOCX) [file pone.0128240.s001.docx]

Hsc70 MS**K**GPAVGIDLGTTYSCVGVFQHG**K**VEIIANDQGNRTTPSYVAFTDTERLIGDAA**K**NQVA 60

Hsp70 MA**K**AAAIGIDLGTTYSCVGVFQHG**K**VEIIANDQGNRTTPSYVAFTDTERLIGDAA**K**NQVA 60

Hsc70 MNPTNTVFDA**K**RLIGRRFDDAVVQSDM**K**HWPFMVVNDAGRP**K**VQVEY**K**GET**K**SFYPEEVS 120

Hsp70 LNPQNTVFDA**K**RLIGR**K**FGDPVVQSDM**K**HWPFQVINDGD**K**P**K**VQVSY**K**GET**K**AFYPEEIS 120

Hsc70 SMVLT**K**M**K**EIAEAYLG**K**TVTNAVVTVPAYFNDSQRQAT**K**DAGTIAGLNVLRIINEPTAAA 180

Hsp70 SMVLT**K**M**K**EIAEAYLGYPVTNAVITVPAYFNDSQRQAT**K**DAGVIAGLNVLRIINEPTAAA 180

Hsc70 IAYGLD**KK**VGAERNVLIFDLGGGTFDVSILTIEDGIFEV**K**STAGDTHLGGEDFDNRMVNH 240

Hsp70 IAYGLDRTG**K**GERNVLIFDLGGGTFDVSILTIDDGIFEV**K**ATAGDTHLGGEDFDNRLVNH 240

Hsc70 FIAEF**K**R**K**H**KK**DISEN**K**RAVRRLRTACERA**K**RTLSSSTQASIEIDSLYEGIDFYTSITRA 300

Hsp70 FVEEF**K**R**K**H**KK**DISQN**K**RAVRRLRTACERA**K**RTLSSSTQASLEIDSLFEGIDFYTSITRA 300

Hsc70 RFEELNADLFRGTLDPVE**K**ALRDA**K**LD**K**SQIHDIVLVGGSTRIP**K**IQ**K**LLQDFFNG**K**ELN 360

Hsp70 RFEELCSDLFRSTLEPVE**K**ALRDA**K**LD**K**AQIHDLVLVGGSTRIP**K**VQ**K**LLQDFFNGRDLN 360

Hsc70 **K**SINPDEAVAYGAAVQAAILSGD**K**SENVQDLLLLDVTPLSLGIETAGGVMTVLI**K**RNTTI 420

Hsp70 **K**SINPDEAVAYGAAVQAAILMGD**K**SENVQDLLLLDVAPLSLGLETAGGVMTALI**K**RNSTI 420

Hsc70 PT**K**QTQTFTTYSDNQPGVLIQVYEGERAMT**K**DNNLLG**K**FELTGIPPAPRGVPQIEVTFDI 480

Hsp70 PT**K**QTQIFTTYSDNQPGVLIQVYEGERAMT**K**DNNLLGRFELSGIPPAPRGVPQIEVTFDI 480

Hsc70 DANGILNVSAVD**K**STG**K**EN**K**ITITND**K**GRLS**K**EDIERMVQEAE**K**Y**K**AEDE**K**QRD**K**VSS**K**N 540

Hsp70 DANGILNVTATD**K**STG**K**AN**K**ITITND**K**GRLS**K**EEIERMVQEAE**K**Y**K**AEDEVQRERVSA**K**N 540

Hsc70 SLESYAFNM**K**ATVEDE**K**LQG**K**INDED**K**Q**K**ILD**K**CNEIINWLD**K**NQTAE**K**EEFEHQQ**K**ELE 600

Hsp70 ALESYAFNM**K**SAVEDEGL**K**G**K**ISEAD**KKK**VLD**K**CQEVISWLDANTLAE**K**DEFEH**K**R**K**ELE 600

Hsc70 **K**VCNPIIT**K**LYQSAGGMPGGMPGGFPGGGAPPSGGASSGPTIEEVD 646

Hsp70 QVCNPIISGLYQGAGG-PG--PGGF--GAQGP**K**GGSGSGPTIEEVD 641

Figure S1. Details of LC-MS/MS analysis for both Hsc70-Ub and Hsp70-Ub as ubiquitinated by UbcH5a and CHIP in vitro. The sequence alignment is annotated with the observed regions in red, all lysines in bold, and ubiquitinated lysines in yellow.
