# Supplementary figures and images for: Biochemical and Proteomic Analysis of Ubiquitination of Hsc70 and Hsp70 by the E3 Ligase CHIP
Source: PLoS One. 2015 May 26;10(5):e0128240. doi: 10.1371/journal.pone.0128240 (PMC4444009; doi:10.1371/journal.pone.0128240)

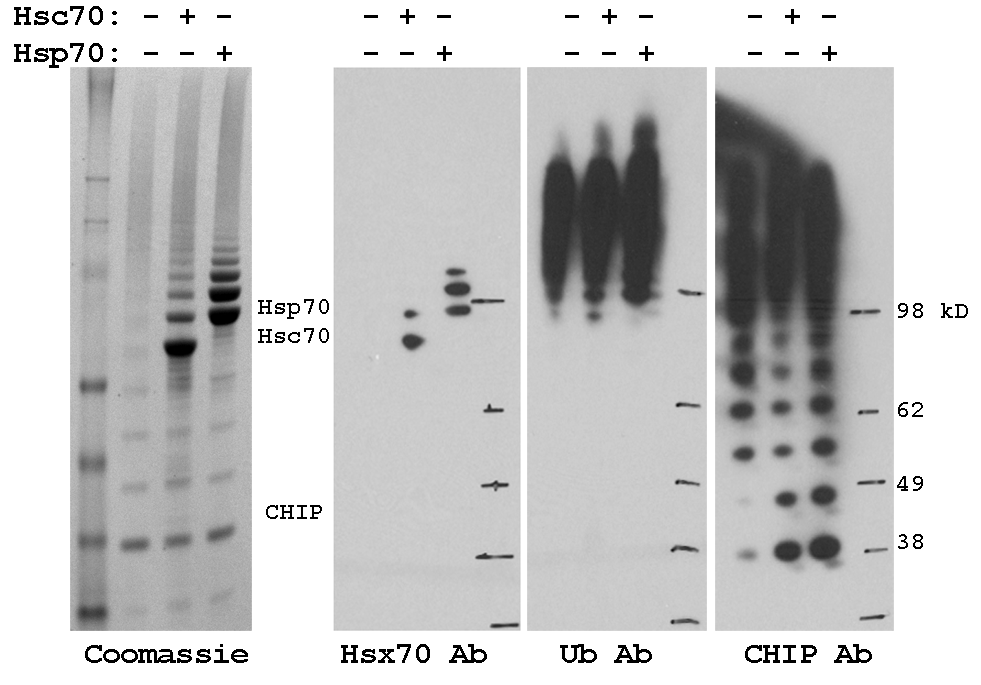

Supplement: S2 Fig — In vitro ubiquitination reactions of 30 minutes without and with substrates were blotted for Hsx70, Ub and CHIP with the respective antibodies as labeled. While high molecular weight species are identified as containing CHIP and Ub, only products containing +1–2 Ub can be observed for the Hsx70 substrates. (TIF) [file pone.0128240.s002.tif]

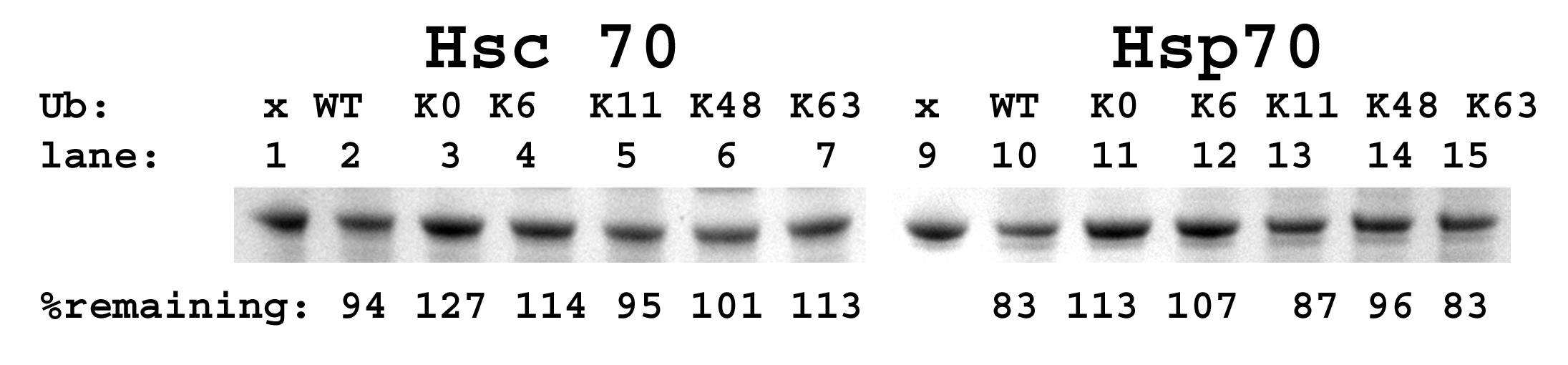

Supplement: S3 Fig — In the same manner as Fig 2, concurrent ubiquitination and degradation reactions with UbcH5a and the Ub type indicated were incubated for 2 hours at 37°C. The amount of unmodified Hsx70 was quantified and the relative amount of protein remaining in each reaction is indicated. (TIF) [file pone.0128240.s003.tif]

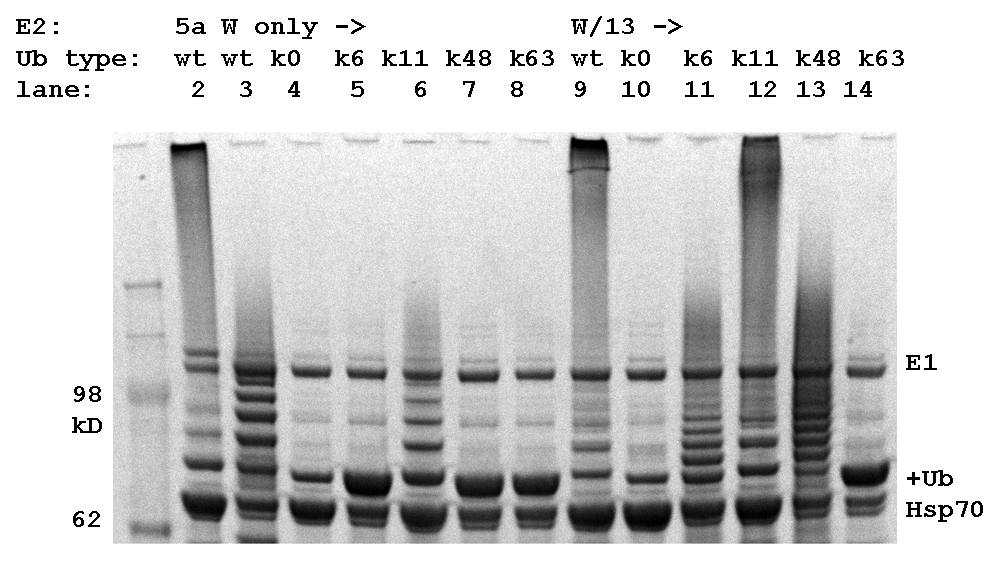

Supplement: S4 Fig — Hsp70 ubiquitination reactions were incubated for 30 minutes as above except using Ube2W alone or in combination with Ubc13/Uev1a the E2. Samples for mass spectrometry proteomics were taken from lanes 3, 4, 7, and 13 as noted in the text. (TIF) [file pone.0128240.s004.tif]

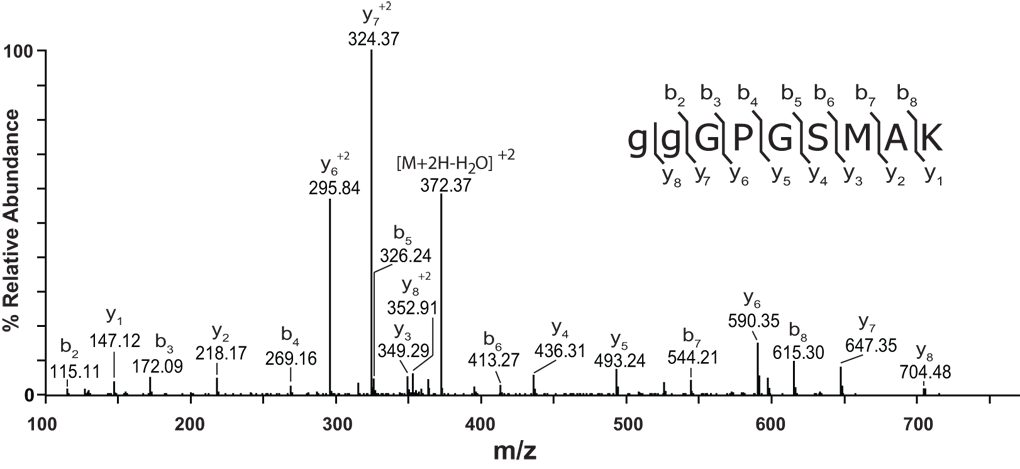

Supplement: S6 Fig — The annotated MS/MS spectrum for the peptide (gg)-GPGSMAK identifies the signature Ub diGly tag attached directly to the N-terminus of the Hsp70 protein. (TIF) [file pone.0128240.s006.tif]
