# Supplementary material for: Biochemical and Proteomic Analysis of Ubiquitination of Hsc70 and Hsp70 by the E3 Ligase CHIP
Source: PLoS One. 2015 May 26;10(5):e0128240. doi: 10.1371/journal.pone.0128240 (PMC4444009; doi:10.1371/journal.pone.0128240)
Supplement: S5 Fig — (DOCX) [file pone.0128240.s005.docx]

Hsp70_w_wt MA**K**AAAIGIDLGTTYSCVGVFQHG**K**VEIIANDQGNRTTPSYVAFTDTERLIGDAA**K**NQVA 60

Hsp70_w_k0 MA**K**AAAIGIDLGTTYSCVGVFQHG**K**VEIIANDQGNRTTPSYVAFTDTERLIGDAA**K**NQVA 60

Hsp70_w_k48r MA**K**AAAIGIDLGTTYSCVGVFQHG**K**VEIIANDQGNRTTPSYVAFTDTERLIGDAA**K**NQVA 60

Hsp70_1.5 MA**K**AAAIGIDLGTTYSCVGVFQHG**K**VEIIANDQGNRTTPSYVAFTDTERLIGDAA**K**NQVA 60

Hsp70_w_wt LNPQNTVFDA**K**RLIGR**K**FGDPVVQSDM**K**HWPFQVINDGD**K**P**K**VQVSY**K**GET**K**AFYPEEIS 120

Hsp70_w_k0 LNPQNTVFDA**K**RLIGR**K**FGDPVVQSDM**K**HWPFQVINDGD**K**P**K**VQVSY**K**GET**K**AFYPEEIS 120

Hsp70_w_k48r LNPQNTVFDA**K**RLIGR**K**FGDPVVQSDM**K**HWPFQVINDGD**K**P**K**VQVSY**K**GET**K**AFYPEEIS 120

Hsp70_1.5 LNPQNTVFDA**K**RLIGR**K**FGDPVVQSDM**K**HWPFQVINDGD**K**P**K**VQVSY**K**GET**K**AFYPEEIS 120

Hsp70_w_wt SMVLT**K**M**K**EIAEAYLGYPVTNAVITVPAYFNDSQRQAT**K**DAGVIAGLNVLRIINEPTAAA 180 Hsp70_w_k0 SMVLT**K**M**K**EIAEAYLGYPVTNAVITVPAYFNDSQRQAT**K**DAGVIAGLNVLRIINEPTAAA 180

Hsp70_w_k48r SMVLT**K**M**K**EIAEAYLGYPVTNAVITVPAYFNDSQRQAT**K**DAGVIAGLNVLRIINEPTAAA 180

Hsp70_1.5 SMVLT**K**M**K**EIAEAYLGYPVTNAVITVPAYFNDSQRQAT**K**DAGVIAGLNVLRIINEPTAAA 180

Hsp70_w_wt IAYGLDRTG**K**GERNVLIFDLGGGTFDVSILTIDDGIFEV**K**ATAGDTHLGGEDFDNRLVNH 240

Hsp70_w_k0 IAYGLDRTG**K**GERNVLIFDLGGGTFDVSILTIDDGIFEV**K**ATAGDTHLGGEDFDNRLVNH 240

Hsp70_w_k48r IAYGLDRTG**K**GERNVLIFDLGGGTFDVSILTIDDGIFEV**K**ATAGDTHLGGEDFDNRLVNH 240

Hsp70_1.5 IAYGLDRTG**K**GERNVLIFDLGGGTFDVSILTIDDGIFEV**K**ATAGDTHLGGEDFDNRLVNH 240

Hsp70_w_wt FVEEF**K**R**K**H**KK**DISQN**K**RAVRRLRTACERA**K**RTLSSSTQASLEIDSLFEGIDFYTSITRA 300 Hsp70_w_k0 FVEEF**K**R**K**H**KK**DISQN**K**RAVRRLRTACERA**K**RTLSSSTQASLEIDSLFEGIDFYTSITRA 300

Hsp70_w_k48r FVEEF**K**R**K**H**KK**DISQN**K**RAVRRLRTACERA**K**RTLSSSTQASLEIDSLFEGIDFYTSITRA 300

Hsp70_1.5 FVEEF**K**R**K**H**KK**DISQN**K**RAVRRLRTACERA**K**RTLSSSTQASLEIDSLFEGIDFYTSITRA 300

Hsp70_w_wt RFEELCSDLFRSTLEPVE**K**ALRDA**K**LD**K**AQIHDLVLVGGSTRIP**K**VQ**K**LLQDFFNGRDLN 360 Hsp70_w_k0 RFEELCSDLFRSTLEPVE**K**ALRDA**K**LD**K**AQIHDLVLVGGSTRIP**K**VQ**K**LLQDFFNGRDLN 360

Hsp70_w_k48r RFEELCSDLFRSTLEPVE**K**ALRDA**K**LD**K**AQIHDLVLVGGSTRIP**K**VQ**K**LLQDFFNGRDLN 360

Hsp70_1.5 RFEELCSDLFRSTLEPVE**K**ALRDA**K**LD**K**AQIHDLVLVGGSTRIP**K**VQ**K**LLQDFFNGRDLN 360

Hsp70_w_wt **K**SINPDEAVAYGAAVQAAILMGD**K**SENVQDLLLLDVAPLSLGLETAGGVMTALI**K**RNSTI 420 Hsp70_w_k0 **K**SINPDEAVAYGAAVQAAILMGD**K**SENVQDLLLLDVAPLSLGLETAGGVMTALI**K**RNSTI 420

Hsp70_w_k48r **K**SINPDEAVAYGAAVQAAILMGD**K**SENVQDLLLLDVAPLSLGLETAGGVMTALI**K**RNSTI 420

Hsp70_1.5 **K**SINPDEAVAYGAAVQAAILMGD**K**SENVQDLLLLDVAPLSLGLETAGGVMTALI**K**RNSTI 420

Hsp70_w_wt PT**K**QTQIFTTYSDNQPGVLIQVYEGERAMT**K**DNNLLGRFELSGIPPAPRGVPQIEVTFDI 480 Hsp70_w_k0 PT**K**QTQIFTTYSDNQPGVLIQVYEGERAMT**K**DNNLLGRFELSGIPPAPRGVPQIEVTFDI 480

Hsp70_w_k48r PT**K**QTQIFTTYSDNQPGVLIQVYEGERAMT**K**DNNLLGRFELSGIPPAPRGVPQIEVTFDI 480

Hsp70_1.5 PT**K**QTQIFTTYSDNQPGVLIQVYEGERAMT**K**DNNLLGRFELSGIPPAPRGVPQIEVTFDI 480

Hsp70_w_wt DANGILNVTATD**K**STG**K**AN**K**ITITND**K**GRLS**K**EEIERMVQEAE**K**Y**K**AEDEVQRERVSA**K**N 540

Hsp70_w_k0 DANGILNVTATD**K**STG**K**AN**K**ITITND**K**GRLS**K**EEIERMVQEAE**K**Y**K**AEDEVQRERVSA**K**N 540

Hsp70_w_k48r DANGILNVTATD**K**STG**K**AN**K**ITITND**K**GRLS**K**EEIERMVQEAE**K**Y**K**AEDEVQRERVSA**K**N 540

Hsp70_1.5 DANGILNVTATD**K**STG**K**AN**K**ITITND**K**GRLS**K**EEIERMVQEAE**K**Y**K**AEDEVQRERVSA**K**N 540

Hsp70_w_wt ALESYAFNM**K**SAVEDEGL**K**G**K**ISEAD**KKK**VLD**K**CQEVISWLDANTLAE**K**DEFEH**K**R**K**ELE 600 Hsp70_w_k0 ALESYAFNM**K**SAVEDEGL**K**G**K**ISEAD**KKK**VLD**K**CQEVISWLDANTLAE**K**DEFEH**K**R**K**ELE 600

Hsp70_w_k48r ALESYAFNM**K**SAVEDEGL**K**G**K**ISEAD**KKK**VLD**K**CQEVISWLDANTLAE**K**DEFEH**K**R**K**ELE 600

Hsp70_1.5 ALESYAFNM**K**SAVEDEGL**K**G**K**ISEAD**KKK**VLD**K**CQEVISWLDANTLAE**K**DEFEH**K**R**K**ELE 600

Hsp70_w_wt QVCNPIISGLYQGAGG-PG--PGGF--GAQGP**K**GGSGSGPTIEEVD 641

Hsp70_w_k0 QVCNPIISGLYQGAGG-PG--PGGF--GAQGP**K**GGSGSGPTIEEVD 641

Hsp70_w_k48r QVCNPIISGLYQGAGG-PG--PGGF--GAQGP**K**GGSGSGPTIEEVD 641

Hsp70_1.5 QVCNPIISGLYQGAGG-PG--PGGF--GAQGP**K**GGSGSGPTIEEVD 641

Figure S5. Details of the LC-MS/MS analysis of Hsc70-Ub when ubiquitinated by Ube2W and different forms of Ub. As in the previous figure, the sequence is annotated with observed regions in red, all lysines in bold, and ubiquitinated lysines in yellow.
